# Tazemetostat in the therapy of pediatric INI1-negative malignant rhabdoid tumors

**DOI:** 10.1038/s41598-023-48774-2

**Published:** 2023-12-07

**Authors:** Klara Vejmelkova, Petra Pokorna, Kristyna Noskova, Anna Faustmannova, Klara Drabova, Zdenek Pavelka, Viera Bajciova, Martin Broz, Pavel Tinka, Marta Jezova, Hana Palova, Leos Kren, Dalibor Valik, Ondrej Slaby, Jaroslav Sterba

**Affiliations:** 1 Department of Pediatric Oncology, University Hospital Brno and Faculty of Medicine, Masaryk University, 62500 Brno, Czechia; 2 Department of Biology, Faculty of Medicine and Central European Institute of Technology, Masaryk University, 62500 Brno, Czechia; 3 Department of Hospital Pharmacy, University Hospital Brno and Faculty of Medicine, Masaryk University, 62500 Brno, Czechia; 4 Department of Pharmacology, Faculty of Medicine, Masaryk University, 62500 Brno, Czechia; 5 Department of Pharmacology and Toxicology, Faculty of Pharmacy, Masaryk University, 62500 Brno, Czechia; 6 Department of Pathology, University Hospital Brno and Faculty of Medicine, Masaryk University, Brno, 62500 Czechia; 7 Department of Laboratory Medicine, University Hospital Brno and Department of Laboratory Methods, Faculty of Medicine, Masaryk University, 62500 Brno, Czechia

**Keywords:** Cancer, Genetics

## Abstract

Rhabdoid tumors are aggressive tumors that may arise in the kidney, soft tissue, central nervous system, or other organs. They are defined by *SMARCB1* (*INI1*) or *SMARCA4* alterations. Often, very young children are affected, and the prognosis is dismal. Four patients with primary atypical teratoid rhabdoid tumor (AT/RT, a rhabdoid tumor in the central nervous system) were treated by resection and high dose chemotherapy. Tazemetostat was introduced after completion of chemotherapy. Three patients have achieved an event free survival of 32, 34, and 30 months respectively. One progressed and died. His overall survival was 20 months. One patient was treated for a relapsed atypical teratoid rhabdoid tumor. The treatment combined metronomic therapy, radiotherapy, tazemetostat and immunotherapy. This patient died of disease progression, with an overall survival of 37 months. One patient was treated for a rhabdoid tumor of the ovary. Tazemetostat was given as maintenance after resection, chemotherapy, and radiotherapy, concomitantly with immunotherapy. Her event free survival is 44 months. Only approximately 40% of patients with rhabdoid tumors achieve long-term survival. Nearly all relapses occur within two years from diagnosis. The event free survival of four of the six patients in our cohort has exceeded this timepoint. Tazemetostat has been mostly tested as a single agent in the relapsed setting. We present promising results when applied as maintenance or add on in the first line treatment.

## Introduction

Rhabdoid tumors are aggressive malignancies that primarily affect children below the age of 3 years. While these tumors can manifest in various anatomical localizations, the kidney, central nervous system (known as atypical teratoid rhabdoid tumor or AT/RT) or soft tissue are the most commonly affected sites^1^.

The development of rhabdoid tumors is associated with the inactivation of the *SMARCB1* gene (also called INI1), which encodes a critical component of the SWI/SNF ATP-dependent chromatin remodeling complex. Less commonly, inactivation of another SWI/SNF complex component, *SMARCA4*, is found^2^. The SWI/SNF complex antagonizes the function of the PCR2 complex, which is responsible for methylating histone H3 at the lysine 27 residue (H3K27me3) leading to the formation of transcriptionally silent chromatin. Inactivation of *SMARCB1* or *SMARCA4* causes dysfunction of the SWI/SNF complex. This complex would normally suppress the expression of *EZH2*, it’s dysfunction therefore leads to overexpression of *EZH2*, which subsequently leads to higher activity of the Polycomb complex 2 of which EZH2 is an important component. This leads to widespread trimethylation of H3K27 and subsequent suppression of gene expression^3^.

Tazemetostat (EPZ-6438) is a highly selective EZH2 inhibitor with antineoplastic activity^4^. It has been studied in the treatment of oncologic diseases in adults such as advanced epithelioid sarcoma^5^ or follicular lymphoma^6^, and is now emerging for the treatment of other INI1-negative tumors.

## Results

From 2019 to the end of 2022 four patients with a primary AT/RT, one patient with a relapsed AT/RT and one patient with a malignant rhabdoid tumor of the ovary were treated by tazemetostat.

Patients 1 to 4, who were diagnosed with localized SMARCB1 mutated AT/RT, underwent treatment with a combination of maximal safe resection and chemotherapy as per the COG ACNS0333 trial protocol^7^. Among these patients, complete remission (CR) was achieved in patients 1 and 3, while patients 2 and 4 attained partial remission (PR). Patient and tumor characteristics are shown in Tables 1, and 2 summarizes the treatment prior to the introduction of tazemetostat.

**Table 1:** Patient and tumor characteristics.

Patient	No 1	No 2	No 3	No 4	No 5	No 6
Primary tu/ relapse	Primary	Primary	Primary	Primary	Relapse	Primary
Age at dg	30 months	5 months	30 months	21 months	29 months	13 years
Localization of tumor	Brain–supratentorial, M0	Brain–supratentorial, M0	Brain–infratentorial, M0	Brain–infratentorial, M0	Metastatic relapse spinal cord	Right ovary
Sex	Female	Male	Male	Female	Male	Female
Histology	AT/RT	AT/RT	AT/RT	AT/RT	AT/RT	Rhabdoid tumor
Subtype	SHH	SHH	SHH	Not known	Not known	Not applicable
Somatic alteration	*SMARCB1* c.157C>T/p.R53* + LOH	*SMARCB1* deletion	*SMARCB1* c.618G>A/p.W206* + *SMARCB1* loss	*SMARCB1* deletion	*SMARCB1* loss	*SMARCA4* loss
Germline alteration	No	No	No	No	*SMARCB1* c.1005del/p.Y335*	*SMARCA4* c.260del/p.D87fs

**Table 2:** Treatment before tazemetostat introduction.

Patient	No 1	No 2	No 3	No 4	No 5	No 6
Surgery	GTR	Partial resect	GTR	Partial resect	No	R1 resection
Radiotherapy	No	No	No	No	whole spine (23,0 Gy) + boost to metastasis (15,0 Gy)	peritoneal cavity (15 Gy) + boost to tumor bed (21,6 Gy)
chemotherapy	COG ACNS0333	COG ACNS0333	COG ACNS0333	COG ACNS0333	MEMMAT	3 × BEP + EpSSG NRSTS 2005

Tazemetostat was introduced after the completion of chemotherapy (dosing details and an overview of the whole treatment in Table 3). Reasons for dose reduction were hematotoxicity in patient 3 and 4 and bromide accumulation in patient 2. Patients 1, and 3 maintained CR, completed two years of tazemetostat treatment and are now in follow-up. Their overall survival (OS) at the last follow-up visit is 32 and 34 months, respectively. Patient 4 maintained PR achieved by resection and chemotherapy, with OS of 30 months at the last follow-up. Patient 2 maintained PR for eight months of tazemetostat treatment (four months full dose, four months reduced dose for toxicity), then progressed. The first event-free survival (EFS1) was 13 months. A second PR was achieved by resection. Tazemetostat was reintroduced in the full dose and given concomitantly with immunotherapy consisting of dendritic cell vaccine and nivolumab. After three months another tumor progression occurred, with EFS2 of 5 months. The patient died of disease progression, with an OS of 20 months.

**Table 3:** Tazemetostat treatment.

patient	No 1	No 2	No 3	No 4	No 5	No 6
Introduction of tazemetostat	1,5 months after last cht	1,5 months after last cht	3 months after last cht	3 months after last cht	2 months after RT	3 months after RT
State of disease at the introduction of tazemetostat	CR	PR	CR	PR	PR	CR
Initial dose	2 × 1200 mg/m^2^	2 × 1200 mg/m^2^	2 × 1200 mg/m^2^	2 × 1200 mg/m^2^	2 × 600 mg/m^2^	2 × 520 mg/m^2^
Dose modification	No	2 × 950 mg/m^2^ for bromide toxicity	2 × 600 mg/m^2^ for hematotoxicity	2 × 520 mg/m^2^ for hematotoxicity	2 × 1200 mg/m^2^ At progression	No
Dose 2nd year	2 × 520 mg/m^2^	Not applicable	2 × 520 mg/m^2^	2 × 520 mg/m^2^	Not applicable	2 × 520 mg/m^2^
State of disease at last FU	Continued CR	Died of tumor progression	Continued CR	Continued PR	Died of tumor progression	Continued CR
Overal survival (months)	32	20	34	30	37	44

Patient 5, who harbored germline SMARCB1 alteration, was treated for relapsed AT/RT. The primary tumor had been in the posterior fossa. It had been treated in another country by gross total resection, adjuvant chemotherapy as per the EU-RHAB protocol^8^ and proton radiotherapy (RT) to the tumor bed (54,0 Gy). Nine months after completing treatment a metastatic relapse in the spinal cord was diagnosed with an EFS1 of 21 months. The subsequent treatment, which was as per the MEMMAT protocol^9^ administered concomitantly with photon RT to the whole spine (23,0 Gy) with a boost to the metastasis (15,0 Gy), led to a second PR. Tazemetostat was introduced two months after RT and given concomitantly with MEMMAT treatment. After 6 weeks, metastatic progression in the posterior fossa was diagnosed with an EFS2 of 5 months. Following another six months, MRI showed regression of spinal metastasis and a slight progression of the posterior fossa lesions. Proton RT was then administered to the whole brain (30,6 Gy) with a boost to the posterior fossa (23,4 Gy). MRI after RT confirmed a third PR. Tazemetostat was continued concomitantly with immunotherapy consisting of nivolumab and ipilimumab. Immunotherapy had to be discontinued after three months due to grade 3 autoimmune colitis. Subsequently, new tumor sites were found in the pelvic region, liver and lungs and the patient died of tumor progression with an EFS3 of 8 months and an OS of 37 months.

Patient 6 was diagnosed with a stage IIB tumor of the right ovary at the age of 13 years. An R1 resection was performed and an ovarian malignant rhabdoid tumor arising in the context of a germline SMARCA4 alteration was confirmed. The patient underwent adjuvant chemotherapy consisting of three cycles of standard BEP, two cycles as per EpSSG NRSTS 2005^10^ (discontinued for grade IV febrile neutropenia) and RT of the peritoneal cavity (15 Gy) with a boost to the tumor bed (21,6 Gy). Tazemetostat maintenance concomitantly with nivolumab was initiated. This treatment lasted for two years with no significant toxicity. At the last follow-up, 10 months after completing the treatment, the patient was in complete remission, with an EFS/OS of 44 months.

## Discussion

Although the introduction of high-dose chemotherapy with RT for AT/RT has significantly increased EFS and OS rates, more than 50% of children diagnosed with AT/RT still die of tumor progression^7^. The young age of most AT/RT patients renders them particularly susceptible to the toxic side effects of RT, thereby adversely impacting the quality of life of survivors.

The four patients with primary AT/RT in our cohort were all younger than three years of age, with one patient younger than one year. Treatment of these patient involved chemotherapy and high-dose chemotherapy according to the COG ACNS0333 protocol. The focal RT for localized disease was omitted in our patients and up to 2 years of tazemetostat maintenance was employed instead. According to the results of ACNS0333, 91% of the progressions occurred within two years from diagnosis and at four years the EFS was 37%^7^. Three of four of our patients demonstrated an EFS ranging from 2,5 to 3 years at last follow-up. The patient treated for a metastatic relapse of AT/RT achieved an OS of 37 months (EFS1 = 21 months, EFS2 = 6 months, EFS3 = 8 months). Long-term survival in relapsed patients is rare, as only 5% of early relapsed patients in the European Rhabdoid Registry survived five years from diagnosis^11^. In such patients, an EFS is mostly counted in weeks rather than months^12^. The two deceased AT/RT patients were less than one year old at presentation, which is a recognized risk factor. Pertaining the molecular stratification of AT/RT, the non-TYR (= SHH or MYC) subtypes have been postulated to exhibit unfavorable prognosis^11^. One of the deceased patients was confirmed to belong to the SHH subtype. Conversely, two surviving patients also presented with the SHH subtype.

In the patient with stage II ovarian rhabdoid tumor the EFS was 44 months. Long term survival for stage II patients is described as around 40% and most relapses occur in the first 2 years, essentially none after 5 years^13^.

Concerning tolerance, the majority of our patients experienced grade 1 or 2 nausea and/or vomiting that did not necessitate dose reduction. Thrombopenia and neutropenia posed greater concern leading to tazemetostat dose reductions. In the relapsed AT/RT patient the combination of metronomic MEMMAT-based peroral chemotherapy and the full dose of tazemetostat was not feasible due to the combined hematologic toxicity. The youngest of our patients experienced grade 2 bromide accumulation accompanied by increased sleepiness, which also resulted in dose reduction. All our patients suffered from repeated respiratory and gastrointestinal infections, with two individuals experiencing one or more herpes zoster infections and hypogammaglobulinemia. An overview of tazemetostat toxicity is shown in Table 4.

**Table 4:** Toxicity of tazemetostat.

patient	No 1	No 2	No 3	No 4	No 5	No 6
Nausea, vomiting	Grade 2	Grade 1	Grade 1	Grade 1	Grade 2	–
Leukocytopenia	–	–	Grade 2	Grade 2	Grade 2	–
Anemia	–	–	Grade 2	Grade 2	–	–
Thrombocytopenia	Grade 2	–	Grade 3	Grade 3	Grade 2	–
hypogammaglobulinemia	Grade 2	–	–	Grade 2	–	–
Herpes zoster	Grade 2	–	Grade 2	–	–	–
Elevated bromide levels	–	Grade 3	–	–	–	–
Elevated liver enzymes	Grade 1	Grade 1	Grade 2	–	Grade 1	–

Despite these encouraging results, international studies involving larger patient cohorts are warranted to comprehensively evaluate the efficacy of tazemetostat in the treatment of pediatric rhabdoid tumors. Rather than employing tazemetostat as a single agent in the relapsed setting, we believe that it should be further explored as maintenance in a sequential manner, or add-on in the first-line treatment, however based on our experience the single agent maintenance seems to be better tolerated.

## Materials and methods

The use of tazemetostat in University Hospital Brno via the Expanded Excess Program was approved by the Ethics Committee of University Hospital Brno. Patients’ parents or guardians have signed informed consent with molecular genetic testing and off-label treatment using tazemetostat. Patients’ parents or guardians have agreed with use of patients’ data for research and publication and signed an informed consent form. All methods were carried out in accordance with relevant guidelines and regulations.

Patients were treated by surgery, chemotherapy, and in some cases radiotherapy (RT) prior to tazemetostat administration, with tazemetostat being added to standard chemotherapy, RT, or metronomic regimens. Tazemetostat was given in dosing recommended by the distributor: 1200 mg/m2 two times a day for AT/RT and 520 mg/m2 two a day for other rhabdoid tumors. Response assessment was conducted using magnetic resonance imaging. Continuous surveillance of adverse effects was undertaken. Adverse events were graded according to CTCAE 5, toxicity grade 3 led to dose modification. Tumor tissue was subjected to histological examination. To investigate the genetic basis of INI1 inactivation and identify potential concurrent alterations, comprehensive molecular profiling using whole-exome sequencing, array CGH, or MLPA was carried out.

## Data Availability

Details of the individual patients’ diagnostics, treatment, and follow up are available from the corresponding author upon reasonable request.
